# Replication Origin Deletion Enhances Poly(3-Hydroxybutyrate-*co*-3-Hydroxyvalerate) Synthesis in Haloarchaea

**DOI:** 10.1128/spectrum.02149-22

**Published:** 2022-10-20

**Authors:** Haibo Yang, Junyu Chen, Ruchira Mitra, Qiong Xue, Hua Xiang, Jing Han

**Affiliations:** a State Key Laboratory of Microbial Resources, Institute of Microbiology, Chinese Academy of Sciences, Beijing, People’s Republic of China; b College of Life Science, University of Chinese Academy of Sciences, Beijing, People’s Republic of China; c International College, University of Chinese Academy of Sciences, Beijing, People’s Republic of China; Shenzhen Bay Laboratory

**Keywords:** haloarchaea, active replication origin, polyploidy, copy number, chromosome, megaplasmid, PHBV synthesis

## Abstract

Although the use of multiple replication origins for chromosome replication has been widely characterized in haloarchaea, whether it is possible to manipulate the chromosome copy number by their genetic engineering is not known, and how it would affect the cell functioning is poorly understood. Here, we demonstrate that deletion of the three active chromosomal origins in Haloferax mediterranei remarkably reduces its DNA amounts and ploidy numbers. Consequently, the mutant strain H. mediterranei Δ123 is more sensitive to UV and mitomycin C. Surprisingly, the cell size increases by 21.2%, and poly(3-hydroxybutyrate-*co*-3-hydroxyvalerate) (PHBV) production in shake flask culture enhances from 7.23 to 8.11 g/L in ΔEPSΔ123, although there is also a decrease in cell growth. In this mutant, the chromosomal copy number decreases, whereas the *pha*-encoding pHM300 megaplasmid copy number increases. Moreover, our transcriptome analysis reveals that the genes involved in primary metabolisms are significantly downregulated in ΔEPSΔ123, whereas those responsible for starch utilization and precursor supplying for PHBV monomers are upregulated. This indicates that more energy and carbon flux is redirected from primary metabolism to PHBV synthesis, thereby enhancing its PHBV accumulation. These findings may therefore provide a rational design to enhance PHBV synthesis by simply tuning the replication origins to modulate the chromosome/megaplasmid copy number ratio and subsequently influence cellular metabolism and physiological functions.

**IMPORTANCE** The haloarchaeon Haloferax mediterranei is a potential producer of PHBV (100% biodegradable plastic) from inexpensive carbon sources. We previously reported that H. mediterranei possessed three active chromosomal origins and, when these origins were deleted, a dormant origin was activated to initiate the replication of chromosome. In this context, in the present study, we first found a close connection between replication initiation and PHBV accumulation. We describe the potential industrial advantages of the strain H. mediterranei ΔEPSΔ123, which includes the enlargement of cell volume by 21.2% and enhancement of PHBV production by 11.2%. We further reveal the possible mechanism that contributes to the greater PHBV production in the ΔEPSΔ123 strain. Overall, we provide here a conceptual advance in the field of synthetic biology by modulating chromosome replication to improve the production of bio-based chemicals.

## INTRODUCTION

DNA replication is one of the most essential processes for all living cells. It starts at certain genomic sites known as origins, where the replication initiator recognizes, binds, and recruits replication machinery components (1, 2). Bacteria usually employ a single origin for chromosome replication, while eukaryotes initiate from multiple origins for the process (2, 3). For archaea, the third domain of life, many species, including haloarchaea, have been characterized to utilize multiple origins for replication initiation (4–9).

The haloarchaeon Haloferax mediterranei is a natural producer of poly(3-hydroxybutyrate-*co*-3-hydroxyvalerate) (PHBV) from inexpensive carbon sources (10). Without supplementing any 3-hydroxyvalerate (3HV) precursor, H. mediterranei can incorporate up to 10 mol% 3HV in the PHBV chain (11). Moreover, due to its adaptability to high-saline conditions, cultivation of H. mediterranei does not require stringent sterilization techniques, and the cells can be easily lysed upon exposure to normal water, thus simplifying the PHBV recovery process. In an attempt to curb down the PHBV production cost, it is important to enhance its production level. In our previous studies, H. mediterranei has been genetically engineered to improve its PHBV production efficiency (12–14). To further facilitate the genomic researches of H. mediterranei, a highly efficient gene knockout system has been developed by our research group (15). The genome of H. mediterranei consists of one chromosome and three megaplasmids (pHM100, pHM300, and pHM500). Most genes involved in PHBV synthesis are located on pHM300 as the *pha* gene cluster (16). Its genome has multiple replication initiator genes (*cdc6*), indicating the presence of multiple replication origins. Consistently, our previous study identified three active replication origins (*oriC1-cdc6A*, *oriC2-cdc6C*, and *oriC3-cdc6G*) and one dormant origin (*oriC4-cdc6H*) in the chromosome of this strain (9). The dormant origin is activated and essentially carries out replication initiation when all the three active origins are deleted. The mutant strain lacking all of the three active origins exhibits a lower growth rate than does the wild type (9).

Polyploidy is a typical and widespread characteristic in haloarchaea. It imparts haloarchaeal species with several advantages, including the long-term survival ability under extreme environmental conditions, a low rate of mutation, genetic redundancy, conferring resistance to desiccation, and also the use of genomic DNA as a phosphate storage polymer (17). Haloarchaea have a wide-ranging and fluctuating chromosome copy number at different growth phases. Most haloarchaea have a higher copy number during the exponential phase. For example, the chromosome copy number of Haloferax volcanii is almost 20 during the exponential phase, and it decreases to 12 copies in the stationary phase (18). In another haloarchaea, Halobacterium salinarum, the chromosome copy number is almost 25 in the exponential phase, and it decreases to 15 copies in the early stationary phase. Consistently, our previous study also found that the copy number of chromosome and pHM300 increases from the lag phase and reaches a maximum at the late exponential phase in H. mediterranei (19). Both copy numbers decrease during the stationary phase. However, the reduction of the chromosomal copy number is greater compared to pHM300. Consequently, the copy number ratio of pHM300 to chromosome increases from the early exponential phase, reaches a maximum in the stationary phase, and then decreases (19). In addition, other researchers have also suggested that culture conditions, such as the phosphate concentration, influence the ploidy level in haloarchaea (20). Since the chromosome copy number is dependent on the replication times per cell cycle, it is proposed that the number of replication origins on the chromosome might be related to the chromosome copy number. Here, a question rises as to whether it is possible to tune the copy numbers of chromosome and megaplasmids in haloarchaea by intervening in the replication process through genetic engineering of the multiple replication origins. Meanwhile, it is important to determine whether any changes in the copy number ratio of chromosome to megaplasmids would affect the cellular adaptation to environment and physiological metabolism, including PHBV synthesis capacity of haloarchaea. Furthermore, it would be interesting to investigate whether the physiological activities can be optimized by genetically manipulating the ploidy level of haloarchaea.

We aim to develop a novel strategy to effectively enhance PHBV production in H. mediterranei by changing its polyploidy level. This study determines the effects of multiple chromosomal origins on the copy number of chromosome and megaplasmids in H. mediterranei and thus on cells’ adaptation to DNA-damaging agents and PHBV synthesis. We demonstrate that genetic manipulation of chromosomal replication origins alters the megaplasmid and chromosomal copy number, which changes the physiological traits of the mutant, ultimately benefitting biopolymer production in haloarchaea. Our analyses show that the weakening of the primary metabolism in the engineered haloarchaea accounts for its enhanced PHBV-producing ability.

## RESULTS

### Multiple replication origins confer high resistance to DNA-damaging conditions.

**FIG 1:**
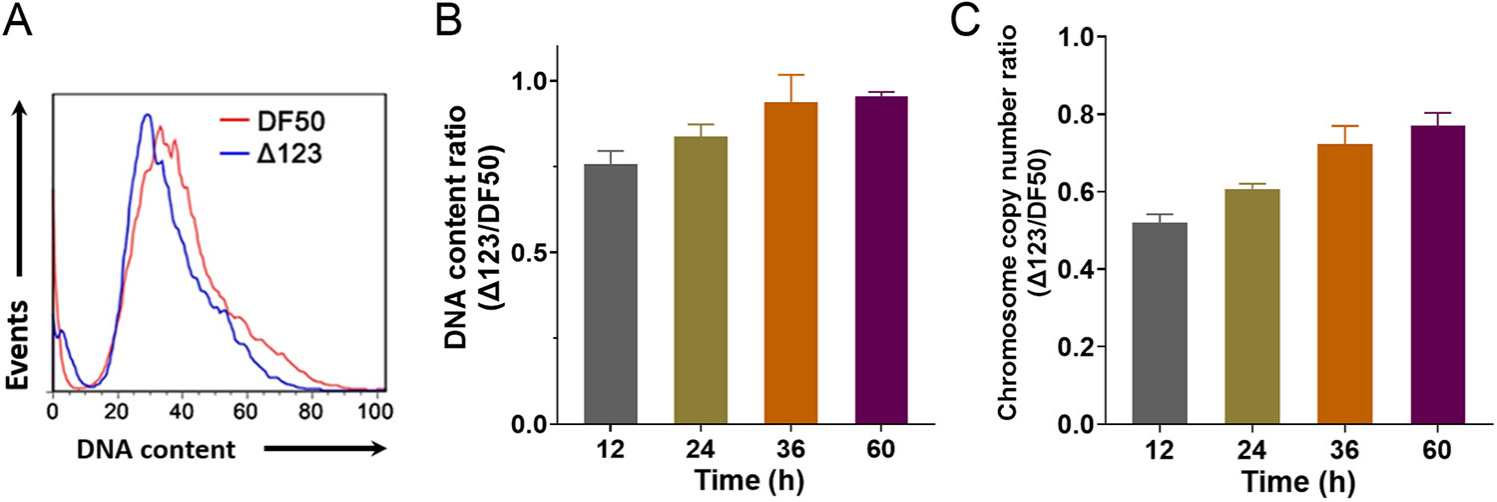
DNA content of H. mediterranei DF50 and Δ123. (A) DNA content of cells measured by flow cytometry. DF50 and Δ123 (DF50Δ*oriC1*Δ*oriC2*Δ*oriC3*) cells cultivated in AS-168 medium for 12 h were fixed with 4% formaldehyde and permeabilized with 70% ethanol. Once stained with acridine orange, the cells were analyzed by flow cytometry. (B) DNA content measured by the diphenylamine colorimetric method. (C) Chromosome copy numbers of cells determined by qPCR. In panels B and C, the AS-168 cultures of DF50 and Δ123 cells at 12, 24, 36, or 60 h were collected and used for DNA content measurement by diphenylamine colorimetric method and chromosome copy number determination by qPCR, respectively. Three biological replicates are represented in panels B and C.

**(i) The DNA content of *H. mediterranei* Δ123 decreases.** The H. mediterranei genome consists of one chromosome and three megaplasmids. The chromosome of H. mediterranei cell has multiple copies. It is speculated that reduction of the multiple replication origins on the chromosome decreased the backup for replication initiation. Thus, deletion of replication origins might have impacted the DNA content of the mutant cell. To check this hypothesis, flow cytometry was first used to determine the DNA content of Δ123 (DF50Δ*oriC1*Δ*oriC2*Δ*oriC3*) and DF50 cultured in AS-168 medium for 12 h. Cells from both strains were dyed with acridine orange and analyzed by flow cytometry immediately. It was observed that the DNA content of Δ123 was less than that of DF50 (Fig. 1A). Moreover, diphenylamine colorimetric method was also used to measure the relative DNA content of Δ123 and DF50. Samples of the two strains were collected at 12, 24, 36, and 60 h based on their growth curves in AS-168 medium. Specifically, for each time point, cultures with a similar optical density at 600 nm (OD_600_) were analyzed. During the entire growth period, the DNA content of Δ123 was less compared to DF50 (Fig. 1B). Moreover, the DNA content ratio of Δ123 to DF50 increased from 0.75 at 12 h (exponential phase) to 0.95 at 60 h (stationary phase), which indicated that the gap between the DNA content of the two strains decreased with cell growth. Quantitative PCR (qPCR) was performed to directly evaluate the chromosome copy numbers of the two strains at the four time points. The chromosome copy number of Δ123 was lower than that of DF50 (Fig. 1C), and the changing pattern of chromosome copy number ratio (Δ123 versus DF50) was consistent with the result of the diphenylamine colorimetric method (Fig. 1B). Thus, it could be concluded that deletion of the three active replication origins in H. mediterranei led to decreased chromosome copy number and DNA content.

**FIG 2:**
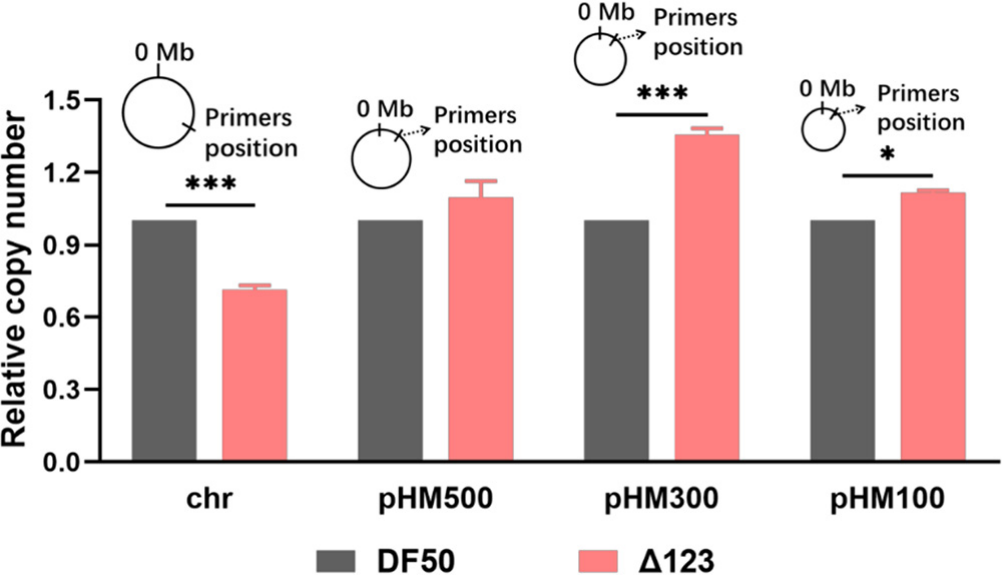
Chromosome copy number of H. mediterranei decreases with active origin deletion. The relative chromosome copy number (chr) and the pHM500, pHM300, and pHM100 copy number of DF50 and Δ123 (DF50Δ*oriC1*Δ*oriC2*Δ*oriC3*) grown in AS-168 medium for 12 h are indicated. Samples were analyzed by qPCR, with three biological replicates. Insets represent the locations of primers used for qPCR on chromosome (chr) and megaplasmids pHM500, pHM300, and pHM100. Statistical significance is indicated by asterisks (*, *P < *0.05; ***, *P < *0.001).

**(ii) Deletion of origins decreases the chromosome copy number of *H. mediterranei*.** There are three active origins on the chromosome of H. mediterranei. qPCR was used to investigate the relationship between the ploidy level and replication origins on the chromosome in the Δ123 mutant. Meanwhile, the influence of three active origin deletions on the copy number of three megaplasmids was analyzed. A pair of primers targeting a fragment on the chromosome, pHM500, pHM300, and pHM100 was designed, respectively (Fig. 2). The exponential phase (12 h) cultures of DF50 and Δ123 in AS-168 medium were collected, and four pairs of primers were used for copy number analysis by qPCR. The relative chromosome copy number of Δ123 to DF50 was 0.71 ± 0.02 (decreased by 29%), whereas they were 1.36 ± 0.03 and 1.12 ± 0.01 for pHM300 and pHM100 (increased by 36% and 12%), respectively (Fig. 2). In contrast, the relative pHM500 copy number of Δ123 to DF50 was 1.09 ± 0.07 and remained at a constant level. Therefore, the results clearly demonstrated that the chromosome copy number remarkably decreased with deletion of the three active replication origins on the chromosome. Surprisingly, the pHM300 and pHM100 copy numbers significantly increased in Δ123 via an unknown mechanism.

**FIG 3:**
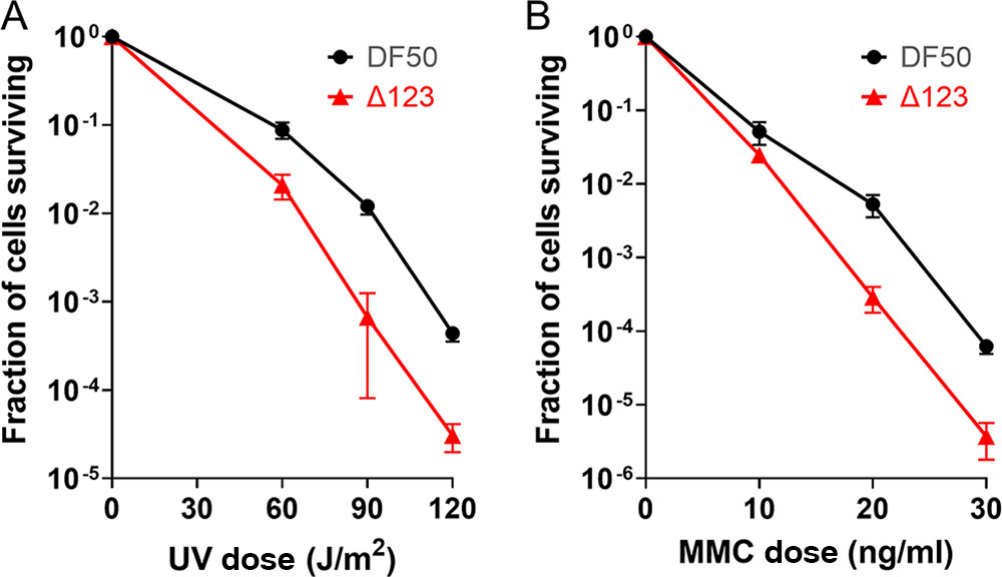
H. mediterranei Δ123 is more sensitive to DNA-damaging agents than is DF50. (A) Cell sensitivity to UV. (B) Cell sensitivity to mitomycin C (MMC). Strain cultures at 12 h were plated on AS-168 plates supplemented with uracil or MMC and exposed to UV. Survivors on AS-168 plates were enumerated after 4 to 5 days of incubation.

**(iii) *H. mediterranei* Δ123 is more sensitive to DNA-damaging agents.** When the three active origins are deleted, a dormant origin *oriC4-cdc6H* is activated and used to replicate the chromosome (9). The pairwise growth competition assay revealed that the growth of H. mediterranei Δ123 was 12.4% slower than that of the control strain DF50 in rich medium. DNA-damaging agents such as UV and mitomycin C lead to double-strand breaks (DSBs). The accurate repair of DSBs relies upon homologous recombination, which is template dependent (21). Since deletion of the multiple replication origins decreased the DNA content and the chromosome copy number, it was likely that the repair process might be less efficient. To better understand the consequences of knocking out origins on cell fitness, we evaluated the cell survival of Δ123 to DNA-damaging agents, UV irradiation, and mitomycin C. With the UV irradiation dose increase from 60 to 120 J/m^2^ and the mitomycin C dose increase from 10 to 30 ng/mL, a significant decrease in survival of DF50 and Δ123 was observed (Fig. 3). Δ123 exhibited an ~10-fold lower tolerance to UV irradiation compared to DF50 under the tested doses (Fig. 3A). Likewise, Δ123 showed a higher sensitivity to mitomycin C compared to the control strain under three different doses (Fig. 3B). The number of Δ123 cells surviving at 30 ng/mL of mitomycin C was almost 50-fold lower than the number of DF50 cells. Our results demonstrated that Δ123 was more sensitive to UV irradiation or mitomycin C than DF50 (Fig. 3). Thus, multiple DNA replication origins of H. mediterranei facilitated the generation of more templates for homologous recombination and conferred higher resistance to DNA-damaging agents. The Δ123 strain contained fewer chromosome copies, thereby leading to a reduced efficiency of DNA repair with homologous recombination.

### Deletion of replication origins improves PHBV production.

**FIG 4:**
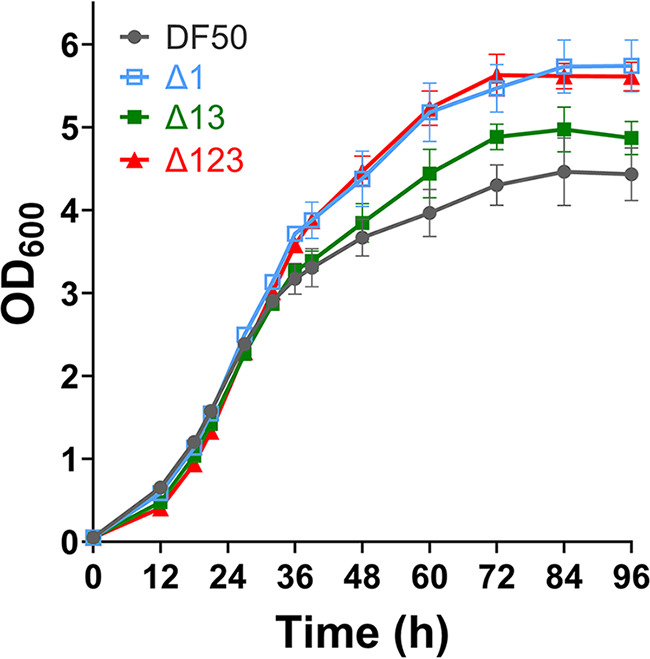
OD_600_ values of H. mediterranei strains with replication origin deletion cultured in AS-168 medium. Δ1, DF50Δ*oriC1*; Δ13, DF50Δ*oriC1*Δ*oriC3*; Δ123, DF50Δ*oriC1*Δ*oriC2*Δ*oriC3*. Three biological replicates are set. The OD_600_ is positively biased by intracellular PHBV granules. The OD_600_ values of the origin knockout mutants are higher than that of the wild type after 36 h.

**(i) Enhanced OD_600_ of origin knockout mutants.** To compare the cell growth of the three origin knockout mutants (Δ1, Δ13, and Δ123) and DF50 in all the growth phases, the four strains were respectively cultivated in AS-168 medium at 37°C. Cell growth was measured by determining the OD_600_ at different stages of cultivation. At the early stages of cultivation, the OD_600_ values of DF50 were similar to those of the three origin knockout mutants. However, the OD_600_ of DF50 was surpassed by the origin knockout mutants at 24 h, and the difference between the mutants and DF50 gradually increased. The ultimate OD_600_ values for Δ13 and Δ1/Δ123 were 11 and 27% higher than that for DF50, respectively (Fig. 4). Thus, although we previously found that the growth rate of DF50 was faster than those of the origin knockout mutants (Δ1, Δ13, and Δ123) in the early exponential phase, as revealed by comparison of the cell number (9), the ultimate OD_600_ of DF50 was much less than that of the origin knockout mutants. This might be because OD_600_ is positively biased by the more intracellular PHBV granules of origin knockout mutants. Based on these results, we proposed that by knocking out the replication origins, the carbon and energy saved from the synthesis of less chromosome might be redirected toward the synthesis of other cellular components, such as PHBV, which resulted in a higher OD_600_.

**FIG 5:**
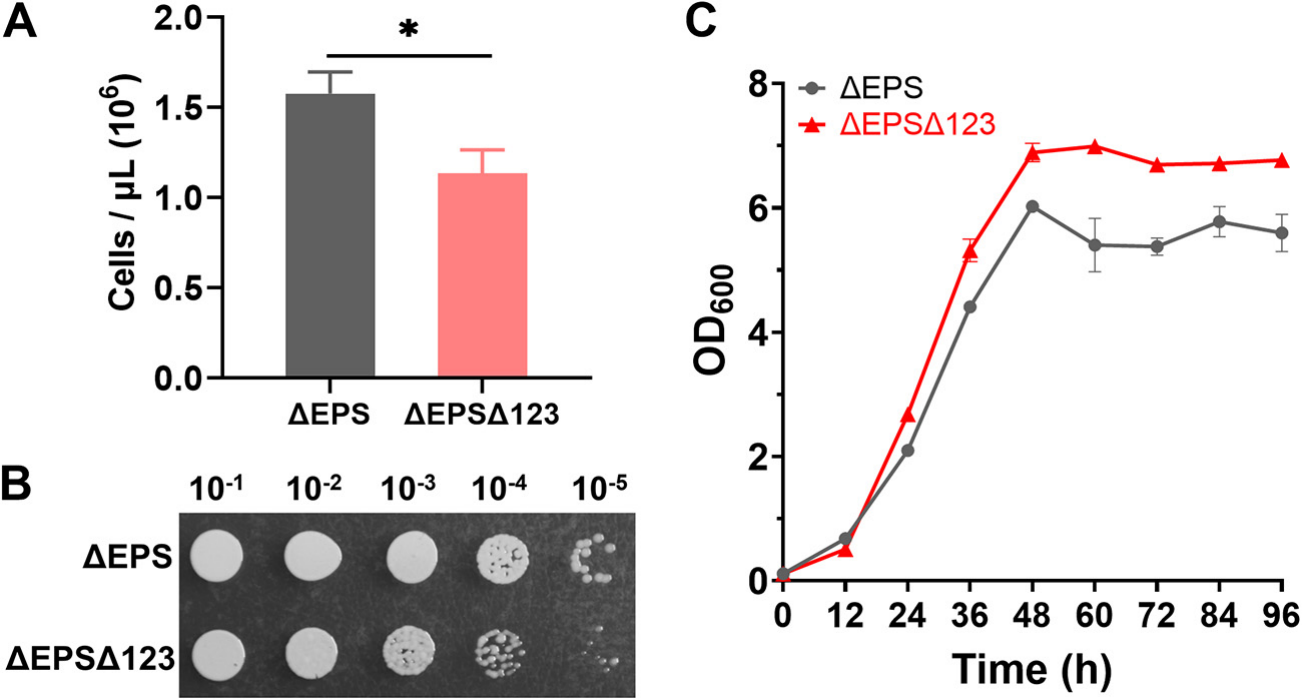
Growth comparison of H. mediterranei ΔEPS and ΔEPSΔ123 cultured in AS-168 medium. (A and B) Total plate count (A) and growth phenotype (B) of ΔEPSΔ123 compared to ΔEPS. Serial dilutions of equal amounts of cells at 24 h were spotted onto AS-168 plates supplemented with uracil. Statistical significance is indicated in panel A by asterisks (***, *P < *0.001). (C) OD_600_ values of ΔEPS and ΔEPSΔ123. The OD_600_ is positively biased by intracellular PHBV granules. Three biological replicates are represented in panels A to C.

**(ii) Decreased growth rate and increased PHBV production in *H. mediterranei* ΔEPSΔ123.** The H. mediterranei ΔEPS strain, with the exopolysaccharide (EPS)-encoding gene cluster deleted, is easy to harvest after fermentation and is the best haloarchaeal strain for PHBV production (12). Therefore, we obtained strain ΔEPSΔ123 (ΔEPSΔ*oriC1*Δ*oriC2*Δ*oriC3*) by knocking out the EPS genes based on Δ123 to evaluate whether the PHBV-producing ability is further improved in ΔEPSΔ123 compared to ΔEPS. We compared the growth rates of ΔEPSΔ123 and ΔEPS strains in AS-168 medium by counting colony numbers because the OD_600_ might be biased by the intracellular PHBV granules. As expected, ΔEPSΔ123 grew slowly compared to ΔEPS, with a growth defect of ~61.7% at 24 h of cultivation in AS-168 medium (Fig. 5A and B). Consistent with the OD_600_ curves of Δ123 versus DF50 (Fig. 4), ΔEPSΔ123 showed a higher OD_600_ than ΔEPS when cultured in AS-168 medium (Fig. 5C). At 24 h, the PHBV content of ΔEPSΔ123 was higher than that of ΔEPS (13.67 wt% versus 7.64 wt%) when cultured in AS-168 medium (which is not favorable for polyhydroxyalkanoate [PHA] synthesis). The PHBV accumulation of ΔEPSΔ123 was 92.3% higher than that of ΔEPS (0.25 g/L versus 0.13 g/L). At 96 h, the final CDW (cell dry weight) and PHBV accumulation of ΔEPSΔ123 reached to 3.74 and 1.15 g/L, respectively, which were 15.79 and 91.67% higher than the corresponding control values (3.23 and 0.6 g/L), respectively (Table 1). These results revealed the reason (i.e., more intracellular PHBV granules) why the origin knockout mutants showed higher OD_600_ values and a greater final CDW but a lower growth rate. Similarly, the other two origin mutants (ΔEPSΔ124 and ΔEPSΔ134) generated by deleting the EPS genes based on Δ124 and Δ134, respectively (9), exhibited higher OD_600_ values than ΔEPS when grown in AS-168 medium (see Fig. S1 in the supplemental material).

**TABLE 1:** PHBV accumulation in H. mediterranei strains cultured in AS-168 medium^*a*^

Phase and strain	Mean ± SD			
	PHBV content (wt%)	3HV fraction (mol%)	CDW^*b*^ (g/L)	PHBV concn (g/L)
24 h, exponential phase				
ΔEPS	7.64 ± 1.50	12.21 ± 0.84	1.72 ± 0.05	0.13 ± 0.04
ΔEPSΔ123^*c*^	13.67 ± 1.47	11.58 ± 0.16	1.80 ± 0.04	0.25 ± 0.04
96 h, stationary phase				
ΔEPS	18.67 ± 0.20	11.69 ± 0.22	3.23 ± 0.14	0.60 ± 0.03
ΔEPSΔ123	30.81 ± 0.03	11.14 ± 0.25	3.74 ± 0.05	1.15 ± 0.02

a AS-168 medium is not favorable to PHBV synthesis. All data are expressed as the means of three biological replicates.

b CDW, cell dry weight.

c ΔEPSΔ123, ΔEPSΔ*oriC1*Δ*oriC2*Δ*oriC3*.

**FIG 6:**
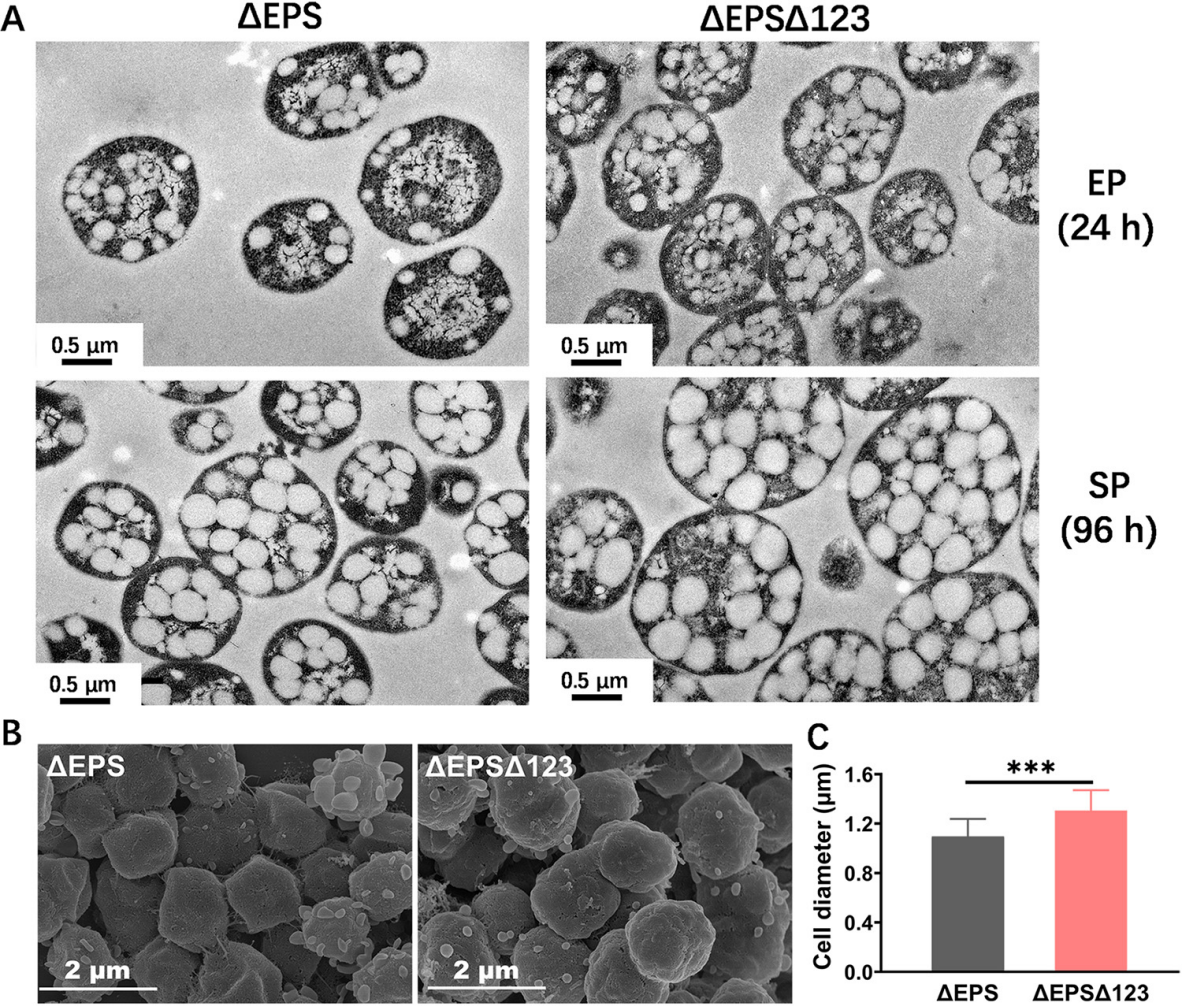
TEM and SEM images of ΔEPSΔ123 and ΔEPS when cultured in MS medium. (A) TEM images of ΔEPS and ΔEPSΔ123 at 24 h (EP, exponential phase) and 96 h (SP, stationary phase). (B) ΔEPS and ΔEPSΔ123 morphology. (C) Average sizes of total 100 cells, measured by ImageJ software. ΔEPS and ΔEPSΔ123 grown in MS medium for 96 h are used for SEM analysis. Statistical significance is indicated in panel C by asterisks (***, *P < *0.001).

Subsequently, we determined the PHBV production of three origin mutants (ΔEPSΔ123, ΔEPSΔ124, and ΔEPSΔ134) and the control strain (ΔEPS) when cultured in fermentation medium (MS medium, which is favorable for PHA synthesis) in a shake flask. The results showed that both ΔEPSΔ123 and ΔEPS reached a much higher CDW and PHBV content in MS medium (Table 2) compared to when they were cultured in AS-168 medium (Table 1). Our previous research showed that the real cell mass (RCM), which equals the CDW minus the PHBV concentration, accurately represented the cell growth rather than CDW when cells produced a large amount of PHA (12). The final RCM of ΔEPSΔ123 (7.05 g/L), ΔEPSΔ124 (6.15 g/L), or ΔEPSΔ134 (7.75 g/L) was significantly lower than that of ΔEPS (8.14 g/L), whereas their CDWs were similar or even lower compared to ΔEPS (Table 2). However, the final PHBV production of ΔEPSΔ123 or ΔEPSΔ124 reached 8.11 or 7.94 g/L, a value that was 11.2 or 9.82% higher than that of ΔEPS, respectively (Table 2). Among the three origin mutants, ΔEPSΔ123 exhibited the strong ability to synthesize PHBV. Subsequently, we further examined the intracellular PHBV granules in ΔEPSΔ123 and ΔEPS by transmission electron microscopy (TEM). At both exponential (24 h) and stationary (96 h) growth phases, ΔEPSΔ123 accumulated more PHBV granules than did ΔEPS (Fig. 6A). This observation was consistent with the PHBV synthesis quantification data. At the stationary growth phase (96 h), the cell size of ΔEPSΔ123 appeared larger than that of ΔEPS (Fig. 6A). Consistent with these observations, further scanning electron microscopy (SEM) showed a 21.2% increase in the mutant cell diameter (1.31 ± 0.17 μm) compared to the control (1.10 ± 0.14 μm) (Fig. 6B and C).

**TABLE 2:** PHBV accumulation in H. mediterranei strains cultured in MS medium^*a*^

Strain	Mean ± SD				
	PHBV content (wt%)	3HV fraction (mol%)	CDW^*b*^ (g/L)	PHBV concn (g/L)	RCM^*c*^ (g/L)
ΔEPS	47.06 ± 0.7	18.19 ± 0.54	15.37 ± 0.25	7.23 ± 0.22	8.14 ± 0.04
ΔEPSΔ123	53.49 ± 1.06	13.86 ± 0.09	15.16 ± 0.12	8.11 ± 0.10	7.05 ± 0.22
ΔEPSΔ124	56.29 ± 2.39	13.18 ± 0.06	14.10 ± 0.58	7.94 ± 0.66	6.15 ± 0.08
ΔEPSΔ134	47.32 ± 0.88	15.91 ± 0.14	14.72 ± 0.11	6.96 ± 0.08	7.75 ± 0.19

a MS medium with 20 g/L starch as the carbon source is favorable to PHBV synthesis. All data are expressed as the means of three biological replicates. Strains: ΔEPSΔ123, ΔEPSΔ*oriC1*Δ*oriC2*Δ*oriC3*; ΔEPSΔ124, ΔEPSΔ*oriC1*Δ*oriC2*Δ*oriC4*; ΔEPSΔ134, ΔEPSΔ*oriC1*Δ*oriC3*Δ*oriC4*.

b CDW, cell dry weight.

c RCM, real cell mass. RCM = CDW − the PHBV concentration.

**FIG 7:**
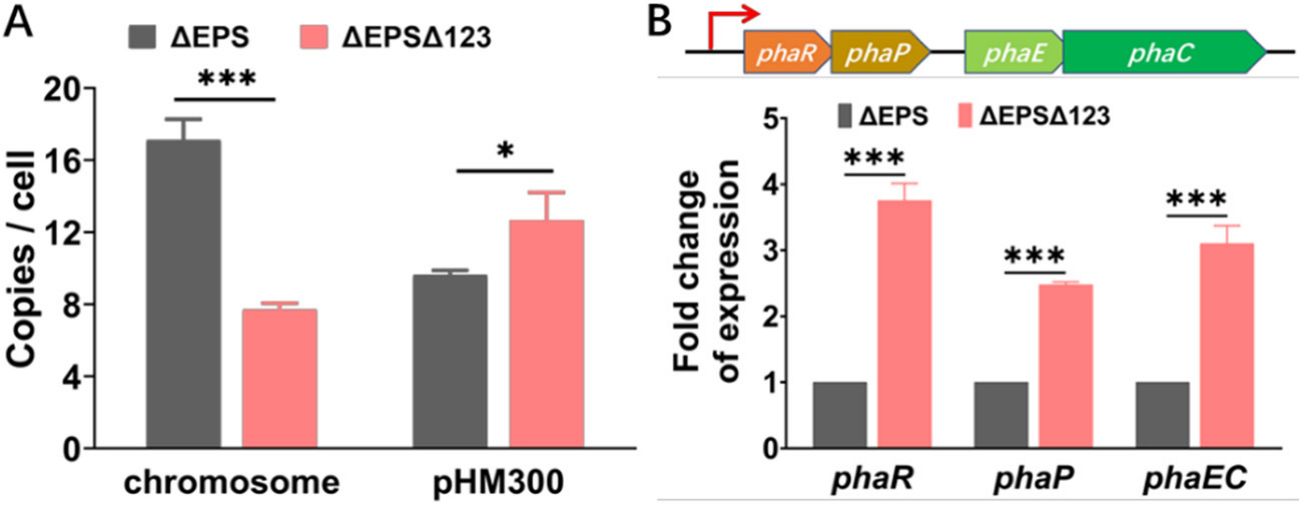
(A) Copy number of chromosome and pHM300 at 24 h determined by qPCR. The *pha* cluster responsible for PHBV synthesis is located on pHM300. Strains were cultured in MS medium. (B) RT-qPCR results of the expression level of *pha* genes (*phaR*, *phaP*, and *phaEC*) in ΔEPS and ΔEPSΔ123 grown in MS medium for 24 h. Statistical significance is indicated by asterisks (*, *P < *0.05; ***, *P < *0.001).

**(iii) Enhanced PHBV production in ΔEPSΔ123 by increasing the gene copy number and the transcription level of the *pha* cluster.** Knocking out of the active replication origins in chromosome led to a decrease in the chromosome copy number and an increase in the pHM300 copy number for H. mediterranei Δ123 when cultured in AS-168 medium (Fig. 2). Since the *pha* gene cluster responsible for PHBV synthesis is located on the pHM300 megaplasmid, we then detected the absolute copy number of pHM300 in strain ΔEPSΔ123 when cultured in MS medium. The culture at 24 h was analyzed by using qPCR. Our results showed a 50.02% decrease in the chromosome copy number and a 31.53% increase in pHM300 megaplasmid copy number in ΔEPSΔ123 compared to ΔEPS (Fig. 7A). This indicated that the copy number of *pha* genes was higher in ΔEPSΔ123 than that in ΔEPS. Simultaneously, reverse transcription-qPCR (RT-qPCR) analysis of the *pha* genes showed that the expression of *phaR*, *phaP*, and *phaEC* were significantly upregulated by 2.75, 1.47, and 2.1, respectively, in ΔEPSΔ123 (Fig. 7B). Therefore, the increase in gene copy number and transcription level of the *pha* cluster validated the increase in PHBV production of ΔEPSΔ123. Based on these results, it may be proposed that the deletion of replication origins led to a decrease in chromosomal copy number and an increase in the pHM300 copy number, which further upregulated the expression of the *pha* genes, leading to the synthesis of more PHBV granules, thus enlarging the cell volume of ΔEPSΔ123.

### Weakened primary metabolism and improved PHBV synthesis pathway in ΔEPSΔ123.

**FIG 8:**
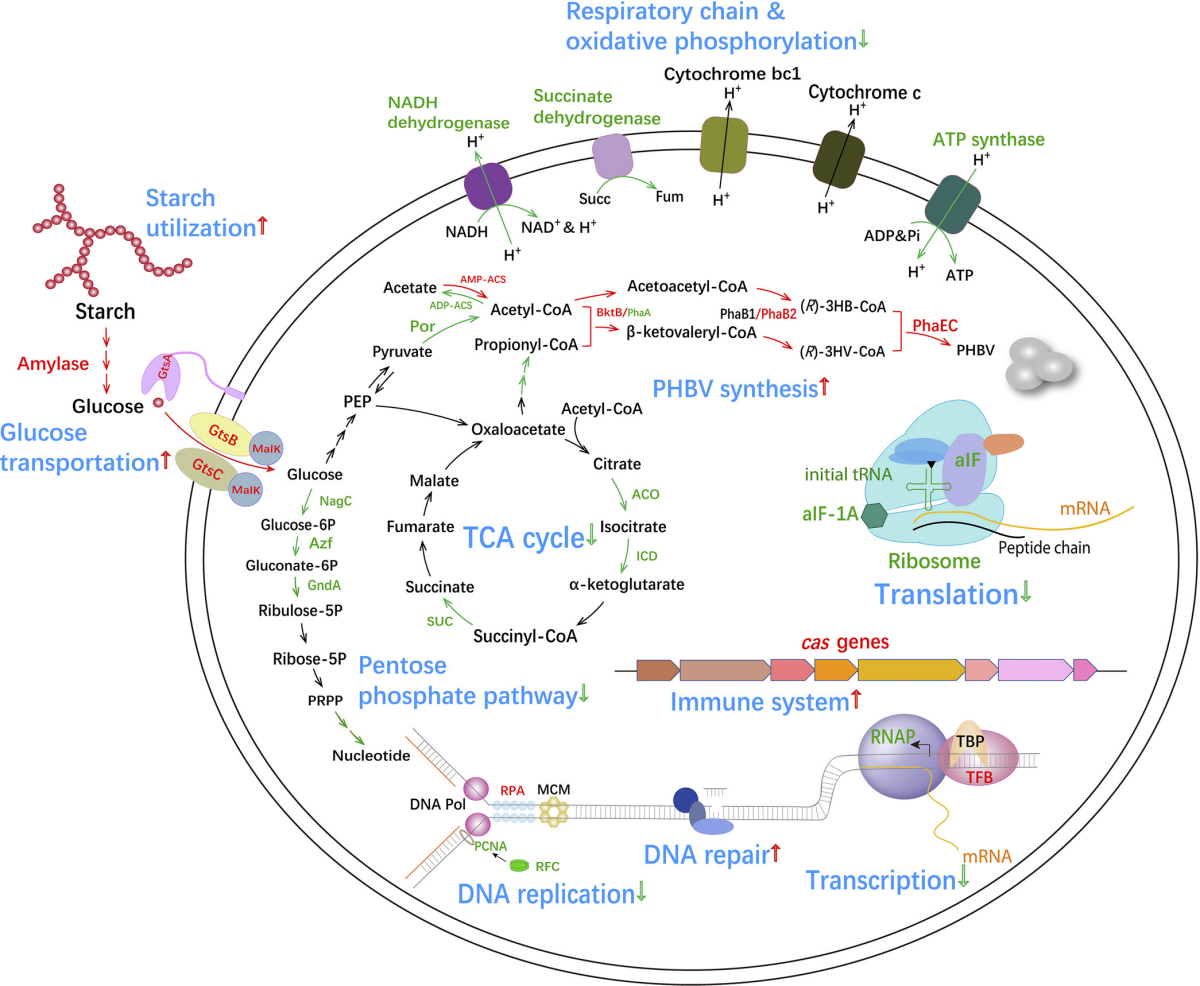
Global effects of the chromosomal replication origins deletion in H. mediterranei. Red and green fonts/arrows indicate upregulation and downregulation of gene expression, respectively. ACO, aconitase; ICD, isocitrate dehydrogenase; SUC, succinyl-CoA synthetase; AMP-ACS, AMP-binding acetyl-CoA synthetase; ADP-ACS, ADP-binding acetyl-CoA synthetase; NagC, glucokinase; Azf, 6-phosphate dehydrogenase; GndA, 6-phosphogluconate dehydrogenase; Por, pyruvate ferredoxin oxidoreductase; BktB/PhaA, β-ketothiolase; PhaB1/PhaB2, β-ketoaceyl-CoA reductase; PhaEC, PHBV synthase; DNA Pol, DNA polymerase; RPA, replication factor A; MCM, DNA helicase; PCNA, DNA polymerase sliding clamp; RFC, replication factor C; RNAP, RNA polymerase; TBP, TATA box-binding protein; TFB, transcription initiation factor B; aIF, archaeal initiation factor; PEP, phosphoenolpyruvate; Succ, succinate; Fum, fumarase. ΔEPS and ΔEPSΔ123 cultivated in MS medium for 24 h were used for the RNA-seq experiment. Three biological replicates of RNA samples of ΔEPS and ΔEPSΔ123 were used for calculation, and a *P*_adj_ value of 0.05 and a log_2_(fold change) of 0.5 are set as the thresholds for significantly differential expression (19).

To gain a deeper insight into the global influence of replication origin knockout on cell genetics and metabolism, we conducted transcriptome sequencing (RNA-seq) and transcriptome analysis of ΔEPSΔ123 versus ΔEPS cultured in MS medium. A total of 650 genes were upregulated, and 496 genes were downregulated significantly [*P < *0.05, |log_2_(fold change)| ≥ 0.5] (see Fig. S2) (22, 23). Such an obvious change in the expression of 1146 genes indicated that the knockout of replication origins had a considerable effect on various processes, including DNA replication and repair, transcription, translation, the CRISPR-Cas system, the quorum sensing system, carbon transportation and metabolism, and energy generation (see Fig. S3, Table S3, and Data Set S1 in the supplemental material). The differentially expressed genes were integrated into a cell map to show the changes in both genetic events and carbon metabolism in ΔEPSΔ123 (Fig. 8).

The expression of initiator Cdc6A recognizing the ORB of *oriC1*, PCNA (DNA polymerase sliding clamp), and RFC (replication factor C) involved in the DNA replication process were generally downregulated in ΔEPSΔ123. In contrast, the expression of DNA excision repair proteins, DNA mismatch repair protein, and resolvase were upregulated. In addition, the genes encoding RNA polymerase (RNAP) were significantly downregulated. Moreover, the genes encoding ribosomal proteins, tRNA synthetases, and translation factors involved in protein translation process were significantly downregulated. Meanwhile, the expression of several enzymes involved in the tricarboxylic acid (TCA) cycle and the pentose phosphate pathway (PPP), including pyruvate-ferredoxin oxidoreductase, aconitate hydratase, isocitrate dehydrogenase, succinyl coenzyme A (succinyl-CoA) synthetase, glucokinase, 6-phosphate dehydrogenase, and 6-phosphogluconate dehydrogenase were downregulated to various degrees. The expression of several respiratory chain complexes and ATP synthases was also downregulated. This implied that the primary metabolism, including DNA replication, RNA transcription, protein translation, energy generation, the TCA cycle, and the PPP, were all weakened. These observations explained the low growth rate of the replication origin deletion mutant. In addition, the *cas* gene cluster (*cas6-cas8b-cas7-cas5h-cas3-cas4-cas1-cas2*) and the gene encoding phage integrase were significantly upregulated, which might impose a fitness cost to the host manifesting a lower growth rate. The expression of 12 gas-vesicle operon proteins was also significantly upregulated.

The genes encoding two amylases, three chitinases, and a glucose transporter composed of GtsA, GtsB, GtsC, and Malk were significantly upregulated. This indicated that starch degradation was boosted in ΔEPSΔ123. However, its degradation product (glucose) accumulated in the fermentation medium of ΔEPSΔ123 (see Fig. S4) due to the downregulation of pyruvate ferredoxin oxidoreductase catalyzing the acetyl-CoA from propionate. Thus, the input of acetyl-CoA, an important precursor for PHBV synthesis, did not increase via the glycolysis pathway. However, the genes encoding the three AMP-forming acetyl-CoA synthetases catalyzing acetate to acetyl-CoA were significantly upregulated, whereas the expression of ADP-forming acetyl-CoA synthetase converting acetyl-CoA to acetate was downregulated. This probably reduced the conversion of acetyl-CoA into acetate and activated more acetate into acetyl-CoA. Furthermore, the weakened TCA cycle and the slow cell growth of ΔEPSΔ123 (Fig. 5A and B) demonstrated that less acetyl-CoA entered into the TCA cycle. Therefore, it was speculated that although the glycolysis pathway was weakened, more acetyl-CoA was channeled toward PHBV biosynthesis in ΔEPSΔ123. Meanwhile, the genes related to PHBV synthesis (*bktB*, *phaE*, and *phaC*) and its regulation (*phaR* and *phaP*) were significantly upregulated in ΔEPSΔ123, a finding in accordance with the RT-qPCR detection result (Fig. 7B). Several genes involved in propionyl-CoA supplying were downregulated, which might have led to a decreased 3HV molar fraction in PHBV. Improved starch utilization efficiency and increased supply of acetyl-CoA from the central carbon metabolism, combined with increased expression of the *pha* gene cluster, resulted in enhanced PHBV accumulation in ΔEPSΔ123.

## DISCUSSION

DNA replication in archaea is usually initiated by multiple replication origins. Haloarchaeal genomes are characterized by the presence of chromosome, minichromosome, and megaplasmids. Phylogenetic and genomic context analyses of many archaeal replication origins suggest that while the most conserved origin *oriC1-cdc6A* is inherited from the ancestor of archaea (24, 25), many other replication origins are acquired by horizontal gene transfer (6, 9, 25). The initiation of the acquired replication origin will thus alter the manner in which the host chromosome is replicated (6). Moreover, the acquired replication origins and accompanying foreign genomic contents may help archaeal cells to adapt to variable environments (25). For acquired dormant replication origins in haloarchaea, they may be active or dormant as a result of different intracellular and extracellular conditions, which may act as an additional adaptive feature (9). Our previous study reports that H. mediterranei has three active and one dormant replication origins on its chromosome. The deletion of three active replication origins activates the dormant origin to initiate the chromosome replication (9). In the present study, we have demonstrated that the multiple chromosomal replication origins confer higher resistance to DNA damage to the H. mediterranei cells. Beyond our expectations, the origins knockout mutant of H. mediterranei yields higher OD_600_ values and higher PHBV accumulation compared to its parent strain. Multiple origin deletion may contribute to the PHBV synthesis in haloarchaea through (i) the decrease in chromosome copy number and the downregulated genes involved in primary metabolism and (ii) the increase in pHM300 copy number and the upregulated *pha* gene cluster in the pHM300 megaplasmid.

In prokaryotes, polyploids are well distributed among the archaeal domain and several bacterial species, including proteobacteria and cyanobacteria. In addition to the cell growth phase, the culture condition has been also reported to influence the chromosomal copy number of haloarchaea (20). Similarly, the chromosomal copy number of H. mediterranei Δ123 is different when cultured in the two different media, i.e., AS-168 and MS medium. Another factor affecting the ploidy level is proposed to be DNA replication origin. In haloarchaea, the multiple origins maintain different initiation efficiencies (17). Therefore, it is speculated that if an origin is initiated more than once in a cycle, the chromosome copy number will be elevated. In contrast, if replication origins initiate replication at lower frequency (e.g., less than once for each cycle), the copy number will be reduced. Likewise, fewer replication origins mean less probability for a chromosome to be replicated, which possibly leads to fewer chromosome copies. Our present study demonstrates that the multiple replication origin is responsible for the higher DNA content and ploidy level in H. mediterranei. Sequential deletion of the active origins reduces the DNA content and chromosome copy number of H. mediterranei. Similarly, deletion of the major and highly conserved archaeal origin, *oriC1*-*orc1*, reduces the chromosome copy number and greatly benefits the cell growth fitness of H. volcanii (26). In contrast, deletion of the less-conserved origin, *oriC2*-*orc5*, drastically increases the chromosome copy number and hindered the growth of H. volcanii (26). The chromosome copy number of haloarchaea shows an evolutionary advantage in desiccation resistance. For example, the phosphate-starved H. volcanii cells are 5-fold more sensitive to desiccation compared to normal cells (20). Likewise, H. mediterranei strains lacking the three active origins are more sensitive to DNA-damaging agents. In both situations, the chromosome copy number of haloarchaea is reduced. Thus, it could be inferred that reduced ploidy level diminishes the genetic advantages toward irradiation and desiccation in haloarchaea. Hawkins et al. propose origins as selfish genetic elements that guarantee their own replication at any cost (27). Based on our results, it is tempting to speculate that multiple origins ensure sufficient DNA synthesis by reducing cellular component (e.g., PHBV) synthesis due to this selfish characteristic. Thus, the fitness of wild-type haloarchaea is greatly improved by its ability to withstand DNA damage. Alternatively, a decrease in chromosome copy number may contribute to the genome manipulation of H. mediterranei, because of fewer wild-type templates for homologous recombination repair. Until now, the *pyrF*-based gene knockout system is the only one tool for the genetic manipulation of H. mediterranei, which is time-consuming (15). A faster and more efficient gene editing tool for H. mediterranei, like the CRISPR-Cas genome editing tool, is still needed.

H. mediterranei is a very proficient strain for industrial PHBV production from inexpensive carbon sources. To further enhance its production level, several strategies have been adopted. H. mediterranei extracellularly produces exopolysaccharide. Thus, the supplied carbon source is distributed among the EPS and PHBV synthesis pathways. Deletion of the EPS gene cluster channels the carbon source toward PHBV synthesis, and the production of 21.28 g/L is obtained in 7-L fermenters by 72-h fed-batch culture (12). Next, deletion of the phosphoenolpyruvate synthetase-like (*pps*-like) gene leads to a 70.46% increase in PHBV production (13). *pps*-like deletion upregulates *phaEC*, *phaR*, and *phaP* gene expression and thus promotes the expression of the PHBV monomer supplying pathway. Moreover, *pps*-like deletion activates the transcription of the three cryptic *phaC* genes in H. mediterranei (28). In a recent study, a CRISPR-engineered H. mediterranei strain has been developed by repressing the citrate synthase genes. This strategy increases the PHBV productivity of the strain by 165% (14). In our present study, we developed another interesting strategy to enhance PHBV synthesis from 7.23 g/L (ΔEPS) to 8.11 g/L (ΔEPSΔ123) at the shake-flask level by genetically manipulating the DNA replication origins in chromosome, but the underlying mechanism for how the decreased ploidy level influences the pHM300 copy number remains to be elucidated. The *pha* gene cluster located on megaplasmid pHM300 responsible for PHBV synthesis is upregulated in ΔEPSΔ123. Interestingly, nearly all of the downregulated genes involved in primary metabolism are located on the chromosome, and their downregulation might be partially caused by the decreased chromosome copy number of ΔEPSΔ123 (see Data Set S1). The upregulation of *pha* genes might mainly result from the increased copy number of pHM300 in the mutant. Thus, the mutant exhibits a prolonged cell cycle and decreased cell growth. Consequently, the carbon and energy saved from less chromosome synthesis and slow cell growth are channeled toward more PHBV synthesis.

We have demonstrated that deletion of the three active chromosomal replication origins decreases the degree of polyploidy in H. mediterranei and increases the sensitivity of mutant cells to DNA damage. Unexpectedly, PHBV production in the origin-deleted mutant is enhanced when the growth rate decreases, possibly because less genetic material synthesis leads to slower cell division, and thus more energy and carbon flux is redirected toward PHBV synthesis. Taken together, the present study finds that multiple origin numbers may contribute to the genetic advantages of haloarchaea by increasing the chromosome copy number. Meanwhile, the genetic manipulation of replication origins of haloarchaea reveals a correlation between the chromosomal copy number and the pHM300 megaplasmid copy number that might be further explored to maximize biopolymer production.

## MATERIALS AND METHODS

### Strains and culture conditions.

H. mediterranei and Escherichia coli strains used in this study are listed in Table S1 in the supplemental material. E. coli JM109 and E. coli JM110 were cultured in lysogeny broth (LB) supplemented with 100 μg/mL ampicillin when needed. For growth rate comparison and seed culture preparation, H. mediterranei strains were cultivated at 37°C in the nutrient-rich medium AS-168 (12). For PHBV accumulation, a seed culture of H. mediterranei was inoculated into AS-168 medium (which is not favorable to PHBV synthesis) or MS medium (fermentation medium with 20 g/L starch as the carbon source) (12) at an initial OD_600_ of 0.1 and then cultivated at 37°C for 96 h. When required, uracil and thymidine were added to the medium at final concentrations of 50 and 40 μg/mL, respectively.

### Mutant construction.

The primers used for mutant construction are summarized in Table S2. The plasmid used to knock out the EPS gene cluster was constructed based on the suicide plasmid pHFX (15). Upstream and downstream fragments with a length of ~600 bp of EPS gene cluster were cloned and inserted into pHFX using a OneStep cloning kit (Yeasen Co., Ltd., China). The transformation of H. mediterranei was performed by the polyethylene glycol-mediated method, as described by Cline et al. (29). Mutants were obtained by using the pop-in/pop-out gene knockout method and confirmed by PCR, as described previously (15).

### DNA damage assay.

For the UV irradiation assay, H. mediterranei cells were first cultivated in AS-168 medium for 12 h (OD_600_ ≈ 0.35), diluted, and then plated on AS-168 plates. Once dried, the cells were exposed to UV irradiation at 60, 90, and 120 J/m^2^, respectively, in a UV cross-linker (Scientz 03-II, China). After UV exposure, the cells were shielded from visible light. For the mitomycin C assay, H. mediterranei cells grown in AS-168 medium for 12 h (OD_600_ ≈ 0.35) were diluted and plated on AS-168 plates supplemented with mitomycin C at final concentrations of 10, 20, and 30 ng/mL, respectively. Survivors on AS-168 plates were counted after 4 to 5 days of incubation. This experiment was performed in triplicates.

### Flow cytometry.

After 12 h of incubation in AS-168 medium, H. mediterranei cells (OD_600_ ≈ 0.35) were first fixed according to previously described procedures (30) and then resuspended in 18% SW plus 10 μg/mL acridine orange. Once stained with acridine orange, the cells were analyzed using an Apogee A40 instrument equipped with a 50-mW 488-nm solid state laser (Coherent, USA) and a 510- to 580-nm bandpass filter as described previously (18). For each time point, 5 × 10^4^ cells were analyzed. Calculations were carried out using FlowJo software.

### Diphenylamine colorimetric method for measuring DNA content.

The DNA content was measured by the diphenylamine colorimetric method as described by Hou et al. (31). Briefly, H. mediterranei cells at different growth time points were collected (~20 mg [wet weight]) and was washed once with 20% NaCl solution. Before adding diphenylamine reagent to the cell pellet, 100 μL of 20% NaCl solution was used to resuspend the cells.

### qPCR and rRT-qPCR.

The primers used are listed in Table S2 in the supplemental material. qPCR was used for chromosome copy number analysis. Standard templates used for standard curve generation were amplified from genomic DNA. The amplification efficiency corresponding to each pair of primers was calculated with the standard curve. The amplification efficiencies of primer pairs used for the qPCR were between 95 and 100%. H. mediterranei cells at the indicated time points were collected and resuspended in basal salt solution (18). Cell suspensions diluted with ddH_2_O were used as the template for qPCR. RT-qPCR was used for gene expression analysis. H. mediterranei cells at 24 h (exponential phase) were collected for total RNA extraction using TRIzol reagent (Invitrogen, USA). After DNase digestion, RNA samples were used for cDNA generation by using random hexamers and the MLV reverse transcriptase (Promega, USA). A KAPA SYBR fast qPCR master mix was used, and the reaction was performed and analyzed using a ViiA 7 real-time PCR system (ABI, USA). The fold change in gene expression was calculated according to a previously described method (32).

### Growth curve, total cell count, and cell growth comparison.

Liquid or solid AS-168 medium was used to compare the growth of the H. mediterranei strains. Seed cultures were inoculated into fresh medium and cultured for 4 days. The OD_600_ values of cultures were measured every 12 h by using a microplate reader (BioTek, USA). Cell cultures at 24 h (early exponential phase) were diluted 1:10^6^, and 100-μL dilutions were coated onto AS-168 plates. After 3 to 4 days of incubation at 42°C, the clone numbers were counted on the plates. Meanwhile, a cell growth comparison was also performed. The AS-168 cultures of tested strains at 24 h were adjusted to equal cell densities and serial dilutions were made. Next, 5-μL portions of 10 to 10^6^ dilutions were spotted onto a AS-168 plate and cultured at 42°C for 4 days.

### PHBV accumulation analysis.

Samples (30 mL) were harvested when cultured in AS-168 medium for 24 h (exponential phase) or 96 h (stationary phase) or in MS medium for 96 h. After overnight lyophilization, ~50-mg portions of cells were collected and esterified by using a mixture containing 97% methanol and 3% H_2_SO_4_ with 1 g/L benzoic acid as an internal standard at 100°C. Quantitative analysis of PHBV was performed by GC6820 gas chromatography (Agilent, USA) as previously described (33).

### TEM and SEM analyses.

H. mediterranei cells cultured in MS medium for 24 h (exponential phase) and 96 h (stationary phase) were used for TEM observation with a JEM-1400 electron microscope (JEOL, Japan). The culture at 96 h was used to perform SEM analysis by using a SU8010 apparatus (Hitachi, Japan). The procedures were performed as previously described (31, 34, 35). Based on the SEM images, the average cell diameter was calculated from 100 cells using ImageJ software.

### RNA-seq.

H. mediterranei ΔEPS or ΔEPSΔ*oriC1*Δ*oriC2*Δ*oriC3* cultivated in MS medium for 24 h was collected for RNA extraction by using TRIzol reagent (Invitrogen, USA). Three repeats are set in our transcriptome experiments. A total of 3 μg of RNA was used for strand-specific library construction using a NEBNext Ultra Directional RNA Library Prep kit for Illumina (NEB, USA). The library preparations were sequenced on an Illumina NovaSeq platform, and 150-bp paired-end reads were generated after cluster generation (Novogene Co., Ltd., China). By removing reads containing adapter, N base, and low-quality reads from raw data, clean data were obtained and further used for all of the downstream analyses. Differential expression analysis of two groups was performed using the DESeq2 R package (1.18.0) (22, 23). The resulting *P* values were adjusted using the Benjamini-Hochberg’s approach for controlling the false discovery rate. A *P*_adj_ value of 0.05 and a log_2_(fold change) of 0.5 were set as the thresholds for significantly differential expression (22).

### Statistical analysis.

Results are presented as means ± the standard errors of three independent replicates. Significant differences were performed by one-way analysis of variance, with statistical significance defined as *P* < 0.05 (*) or *P* < 0.001 (***).

### Data availability.

The RNA-seq data have been deposited in the China National Microbiology Data Center and raw data can be available by imputing the accession numbers (NMDC40005706-NMDC400057011) via https://www.nmdc.cn.
