# Supplementary material for: Replication Origin Deletion Enhances Poly(3-Hydroxybutyrate-co-3-Hydroxyvalerate) Synthesis in Haloarchaea
Source: Microbiol Spectr. 2022 Oct 20;10(6):e02149-22. doi: 10.1128/spectrum.02149-22 (PMC9769981; doi:10.1128/spectrum.02149-22)
Supplement: Supplemental file 1 — Tables S1 to S3 and Fig. S1 to S4. Download spectrum.02149-22-s0001.pdf, PDF file, 0.6 MB [file spectrum.02149-22-s0001.pdf]

Supplementary Information

Replication Origin Deletion Enhances the Poly(3-hydroxybutyrate-*co*-3-hydroxyvalerate) Synthesis in Haloarchaea

Haibo Yang[1,2,#], Junyu Chen[1,#], Ruchira Mitra[1,3], Qiong Xue[1,2], Hua Xiang[1,2,*], Jing Han[1,2,*]

[1] State Key Laboratory of Microbial Resources, Institute of Microbiology, Chinese Academy of Sciences, Beijing, 100101, China

[2] College of Life Science, University of Chinese Academy of Sciences, 100049, Beijing, People's Republic of China

[3] International College, University of Chinese Academy of Sciences, 100049, Beijing, People's Republic of China

[*]Correspondence: hanjing@im.ac.cn (for J. Han) or xiangh@im.ac.cn (for H. Xiang)

[#] Authors equally contributed to this work.

Glucose concentration measurement

A total of 100 μL culture at a specific time was centrifuged to remove cells from the medium. The concentration of glucose in the collected supernatant was determined enzymatically by SBA-40C biosensor analyzer (Institute of Biology, Shandong Academy of Sciences, China).

Table S1. Strains used in this study.

Strains	Relevant characteristics	Source or reference
Escherichia coli JM109	*recA1 supE44 endA1 hsdR17 gyrA96 relA1 thi*	(1)
E. coli JM110	*dam dcm* mutant of *E. coli* JM109	Novagen
DF50	*pyrF*-deleted mutant of wild-type strain *H. mediterranei* ATCC 33500	(2)
DF50Δ*oriC1*Δ*oriC2*Δ*oriC3*	*oriC1*, *oriC2* and *oriC3* deletion mutant of DF50	(3)
ΔEPS	EPS synthesis gene cluster deletion mutant of DF50	(4)
ΔEPSΔ*oriC1*Δ*oriC2*Δ*oriC3*	*oriC1*, *oriC2* and *oriC3* deletion mutant of ΔEPS	This study
ΔEPSΔ*oriC1*Δ*oriC2*Δ*oriC4*	*oriC1*, *oriC2* and *oriC4* deletion mutant of ΔEPS	This study
ΔEPSΔ*oriC1*Δ*oriC3*Δ*oriC4*	*oriC1*, *oriC3* and *oriC4* deletion mutant of ΔEPS	This study

Table S2. Primers used in this study.

Primers	Sequence (5’-3’)	Description
HmchrST-F	GCAAACAGACCGCCAATGA	Synthesis of the qPCR standard template for chromosome
HmchrST-R	TCATCTTACGGCGGGAAT	
HmchrCN-F	GGTGATGGTGGGCTACATTA	Detection of chromosome copy number
HmchrCN-R	GGAACAACAACAGCGAGAAC	
HmpHM500ST-F	AACGACGCCAAATCCTCTG	Synthesis of the qPCR standard template for pHM500
HmpHM500ST-R	ACGGTCTTGTACGCCCTGT	
HmpHM500CN-F	GCGTTACGAAGTGTTGTGG	Detection of pHM500 copy number
HmpHM500CN-R	TGGCTGAGTCTGAGGCATA	
HmpHM300ST-F	GTTCTTCAAGCCGTGGTCC	Synthesis of the qPCR standard template for pHM300
HmpHM300ST-R	GTCGACCCGCTTCTCGGTGA	
HmpHM300CN-F	CTCCGCTTCTGTCTCTCCCTCTAC	Detection of pHM300 copy number
HmpHM300CN-R	ACGACGAACTCGCCCAAATCATC	
HmpHM100ST-F	CTCTCGGAGTGCATCTCG	Synthesis of the qPCR standard template for pHM100
HmpHM100ST-R	CAGGCGAAGATCCGTTCG	
HmpHM100CN-F	CTCTCGGAGTGCATCTCG	Detection of pHM100 copy number
HmpHM100CN-R	GAGGCGTTCGGAATGCAC	
qRTphaR-F	GACGAACGACTCAAACGATGC	qRT-PCR analysis of *phaR* gene
qRTphaR-R	GATGAGTCTCCAAACCGCGGT	
qRTphaP-F	GGACGCGATGGACGAGCAGTT	qRT-PCR analysis of *phaP* gene
qRTphaP-R	TCACACCAGACACGCCGGAGT	
qRTphaEC-F	CTCGTCGAACTCGAACGGAGA	qRT-PCR analysis of *phaEC* gene
qRTphaEC-R	CCGACCGATACCTCCGACACG	
EPS-UF	GCAAATATTGCACCGCCAAC	Amplification of upstream flanking regions of EPS gene cluster
EPS-UR	ACCTGTCACTAGTACAATACTAAACTATC	
EPS-DF	AGGCACGACACACTGGAAT	Amplification of downstream flanking regions of EPS gene cluster
EPS-DR	GGTGGCGCTCGATCAGTC	

Table S3. Partial differential gene expression of ΔEPSΔ123 *vs.* ΔEPS.

Pathway	Location	Locus tag	Log_2 (fold change)	*padj*	Gene
starch utilization	chr	HFX_0535	1.477719	2.13E-17	*amyP1*
	chr	HFX_1044	0.637767	1.68E-06	*amy3*
	chr	HFX_1802	0.603223	1.12E-06	*amy4*
	chr	HFX_1803	0.843555	7.50E-12	*amy5*
	pHM100	HFX_4060	2.234116	2.31E-55	*amyP2*
	pHM500	HFX_6123	3.598593	3.61E-144	*amy7*
glucose transporter	chr	HFX_2705	2.019294	1.06E-36	*MalK*
	chr	HFX_2893	1.712237	6.91E-28	*upgC3*
	chr	HFX_2894	1.842403	3.18E-29	*ugpE*
	chr	HFX_2895	2.027869	9.64E-31	*ugpA*
	chr	HFX_2896	1.547569	2.15E-17	*GtsA*
	pHM300	HFX_5025	0.761812	4.15E-05	*upgC3*
	pHM500	HFX_6193	2.961718	3.00E-34	*msmK*
PHBV synthesis	chr	HFX_1023	-0.58632	0.000120432	*phaAα*
	pHM300	HFX_5215	1.266708	1.72E-19	*phaB2*
	pHM300	HFX_5220	1.076388	4.86E-14	*phaE*
	pHM300	HFX_5221	0.991831	7.87E-12	*phaC*
	pHM500	HFX_6003	1.117589	3.21E-16	*bktBβ*
	pHM500	HFX_6004	0.926731	1.03E-15	*bktBα*
TCA cycle	chr	HFX_0511	-1.4212	5.91194E-27	*acnA*
	chr	HFX_2471	-0.6059	8.62519E-05	*sucD*
	chr	HFX_2613	-0.69186	0.00000168	*icd*
nucleotide synthesis	chr	HFX_0010	-1.17574	2.03E-09	*purO*
	chr	HFX_0318	-1.0778	4.05E-08	*pyrE*
	chr	HFX_0370	-0.6274	4.64E-05	*nagC*
	chr	HFX_0485	-1.05674	5.00E-10	*azf*
	chr	HFX_0590	1.697402	8.52E-10	*dgt*
	chr	HFX_1046	0.559853	0.008355849	*purL*
	chr	HFX_1073	-0.73357	0.0000484	*apt2*
	chr	HFX_1282	0.640738	0.00000654	*guaB*
	chr	HFX_1478	-0.65625	0.000000412	*pmm3*
	chr	HFX_1902	-0.84712	4.75E-10	*gndA*
	chr	HFX_1984	-0.53444	0.005670035	*ham1*
	chr	HFX_2043	-0.70575	0.0000227	*nudF*
	chr	HFX_2091	-0.66386	0.000104664	*nudF*
	chr	HFX_2296	-0.76504	0.00395072	*apt3*
	chr	HFX_2751	-0.70301	0.00000477	*ndk*
	chr	HFX_2983	-1.28731	1.86E-18	*cpsG*
	pHM500	HFX_6013	0.641137	0.002029085	*nrd*
	chr	HFX_0001	-3.86271	2.02E-117	*cdc6A*

Pre-replication complex	chr	HFX_2140	-0.51617	0.057179472	*cdc6H*
MCM	chr	HFX_0921	3.561528	9.68E-74	*mcm2*
	chr	HFX_2950	-0.58744	2.90E-06	*mer3*
RPA	chr	HFX_0279	0.600903	2.98E-07	*rpaA*
PCNA	chr	HFX_0176	-0.53164	0.000866644	*dnaN*
RFC	chr	HFX_0154	-0.89825	5.81E-07	*rfcC1*
	chr	HFX_2437	-0.75412	6.85E-09	*rfcB*
	chr	HFX_0205	-0.87966	8.68E-11	*rfcC2*
DNA ligase	pHM500	HFX_6206	-0.81059	1.08E-05	*DNA ligase (NAD+)*
Immune system	pHM500	HFX_6314	2.343453	6.34E-55	*cas6*
	pHM500	HFX_6315	2.18128	3.67E-33	*Cas8*
	pHM500	HFX_6316	2.298326	1.67E-31	*cas7b*
	pHM500	HFX_6317	2.608396	7.64E-29	*cas5b*
	pHM500	HFX_6318	2.402131	1.65E-28	*cas3*
	pHM500	HFX_6319	1.950249	5.50E-15	*cas4*
	pHM500	HFX_6320	1.739989	2.05E-16	*cas1b*
DNA repair	chr	HFX_1430	13.60056	3.70E-29	*dinG*
	chr	HFX_1434	12.22644	1.09E-23	*DNA mismatch repair protein*
	chr	HFX_0921	3.561528	9.68E-74	*mcm2*
	chr	HFX_0116	0.898747	2.17E-08	*recA*
	chr	HFX_2947	0.661598	4.63E-02	*topA*
	pHM500	HFX_6012	0.822655	0.008043128	*xthA*
TBP	chr	HFX_1809	0.512939	0.014555538	*TBP2*
	chr	HFX_1819	-0.81554	2.07E-07	*TBP3*
	pHM300	HFX_5297	0.754473	0.004947967	*TBP4*
TFB	chr	HFX_0761	0.508427	0.007677453	*tfb3*
	pHM100	HFX_4066	1.166408	0.115172439	*tfb8*
	pHM100	HFX_4102	1.099485	0.00035223	*tfb9*
	pHM300	HFX_5177	1.007256	1.51E-08	*tfb10*
RNAP	chr	HFX_0625	-0.82742	0.001233229	*RNAP*
	chr	HFX_0404	-0.50848	0.064338859	*RNAP*
Translation	chr	HFX_0127	-1.04782	2.10E-16	*rpl31R*
	chr	HFX_0128	-1.00474	2.60E-21	*eif6*
	chr	HFX_0129	-1.27448	4.94E-12	*rplX*
	chr	HFX_0146	-1.18774	3.32E-08	*eif1A1*
	chr	HFX_0258	-1.25409	1.69E-16	*sua5*
	chr	HFX_0341	-0.75892	1.94E-05	*rps7*

	chr	HFX_0343	-0.68327	7.68E-07	*tef2*
	chr	HFX_0346	-1.14134	2.98E-09	*tef1A*
	chr	HFX_0347	-1.38428	1.35E-17	*rps10P*
	chr	HFX_0450	-1.31169	2.98E-14	*rplP*
	chr	HFX_0626	-1.04705	1.66E-12	*rpl37AR*
	chr	HFX_0658	-0.88333	3.02E-08	*eif2A*
	chr	HFX_0659	-0.95488	2.17E-09	*rps27E*
	chr	HFX_0660	-1.38613	1.16E-11	*rpl44E*
	chr	HFX_1468	-0.86687	7.65E-08	*rpsJ*
	chr	HFX_1476	-0.88774	3.57E-09	*rbsA*
	chr	HFX_1880	-1.98263	1.52E-33	*rps6E*
	chr	HFX_1941	-1.66709	1.86E-31	*rps19R*
	chr	HFX_1987	-1.27728	7.96E-23	*rps24E*
	chr	HFX_1992	-0.94946	1.46E-13	*eif2G*
	chr	HFX_2041	-0.71151	7.63E-06	*tif1_1*
	chr	HFX_2104	-1.04079	4.83E-13	*prt*
	chr	HFX_2310	-1.18725	7.10E-17	*eif5A*
	chr	HFX_2381	-1.43607	3.16E-24	*rps8A*
	chr	HFX_2732	-1.68562	2.09E-12	*rpl37E*
	chr	HFX_2748	-0.85815	7.06E-09	*rpl7AE*
	chr	HFX_2749	-0.6038	3.52E-07	*rps28E*
	chr	HFX_2750	-0.65623	7.40E-06	*rpl24A*
	chr	HFX_2760	-0.62397	2.57E-07	*rpl21R*
	chr	HFX_2763	-0.84503	3.86E-07	*tef1B*
	chr	HFX_2770	-1.42871	9.62E-32	*rplK*
tRNA synthetase	chr	HFX_0147	-1.00328	2.06E-09	*tyrS*
	chr	HFX_0208	-0.61219	5.47E-07	*alaS*
	chr	HFX_0309	-0.99042	3.57E-15	*argS*
	chr	HFX_0640	-0.99719	8.73E-11	*aspS*
	chr	HFX_0845	-0.72896	1.17E-08	*proS*
	chr	HFX_1134	-0.60786	6.52E-06	*cysS*
	chr	HFX_2014	-0.80047	1.42E-09	*serS*
	chr	HFX_2938	-0.67539	3.96E-09	*valS*
	chr	HFX_2941	-0.56363	7.04E-06	*pheS*
	chr	HFX_2945	-0.826	2.81E-07	*trpS*
Oxidative phosphorylation	chr	HFX_0296	-1.10438	1.02E-20	*atpH*
	chr	HFX_0297	-1.02432	6.84E-18	*atpI*
	chr	HFX_0298	-1.13725	2.01E-12	*atpK*
	chr	HFX_0301	-0.63867	0.000215387	*atpF*
	chr	HFX_0302	-0.74867	0.00000219	*atpA*
	chr	HFX_0303	-1.05582	6.39E-11	*atpB*
	chr	HFX_0305	-1.2463	1.25E-18	*atpD*
	chr	HFX_0428	1.484044	4.94E-22	*cydB*

	chr	HFX_0429	1.613369	1.12E-29	*cydA*
	chr	HFX_0811	-0.7611	0.00000304	*petD*
	chr	HFX_0944	-0.57962	0.000265782	*cbaA*
	chr	HFX_0977	-0.63879	7.61E-08	*nuoB*
	chr	HFX_0979	-0.57277	0.0000348	*nuoH*
	chr	HFX_0980	-0.7707	4.8E-09	*nuoI*
	chr	HFX_0981	-0.90308	1E-11	*nuoJ1*
	chr	HFX_0982	-0.83221	0.000000197	*nuoJ2*
	chr	HFX_0983	-0.82724	6.99E-09	*nuoK*
	chr	HFX_0984	-0.50136	0.001101049	*nuoL*
	chr	HFX_1010	-0.77783	0.00000052	*ctaB*
	chr	HFX_1150	-0.59138	0.0000535	*coxC*
	chr	HFX_1637	-1.00896	4.28E-18	*ndh*
	chr	HFX_1732	1.578892	7.74E-13	*coxA2*
	chr	HFX_1733	2.805046	5.99E-24	*coxB4*
	chr	HFX_2636	-1.02034	7.17E-11	*petA*
	pHM300	HFX_5106	-1.70431	2.33E-24	*narC*
Chitin utilization	pHM300	HFX_5036	2.167658	2.14E-07	*chiA*
	pHM300	HFX_5037	2.846886	8.00E-09	*chiB*
	pHM300	HFX_5119	1.733005	2.78E-33	*chiA3*
Acetate and acetyl-CoA	chr	HFX_0870	1.541805	4.73E-21	*AMP-acs*
	chr	HFX_0998	-1.21341	2.11E-21	*ADP-acs*
	chr	HFX_1371	-0.79207	4.82E-07	*porA*
	chr	HFX_1571	1.668315	8.44E-25	*ilvN*
	chr	HFX_1572	1.696166	3.30E-34	*ilvB*
	chr	HFX_1643	0.794941	5.36E-06	*acs*
	chr	HFX_2150	2.444302	1.31E-46	*acs4*
	pHM300	HFX_5131	2.982071	1.41E-40	*acs*
	pHM500	HFX_6032	5.362638	1.57E-152	*poxB*
Gas vesicle	chr	HFX_1693	1.656685	6.91E-16	*gvpO*
	chr	HFX_1694	1.451594	5.00E-11	*gvpN*
	chr	HFX_1695	1.402356	3.65E-09	*gvpC*
	chr	HFX_1697	1.080507	1.94E-16	*gvpD*
	chr	HFX_1698	0.99075	3.94E-10	*gvpE*
	chr	HFX_1699	0.74841	7.97E-09	*gvpF*
	chr	HFX_1700	0.76377	0.001068418	*gvpG*
	chr	HFX_1701	0.823084	2.29E-06	*gvpH*
	chr	HFX_1702	0.715232	0.000447317	*gvpI*
	chr	HFX_1703	0.679182	0.000397755	*gvpJ*
	chr	HFX_1705	0.723248	5.61E-07	*gvpL*
	chr	HFX_1706	0.692051	0.008277124	*gvpM*

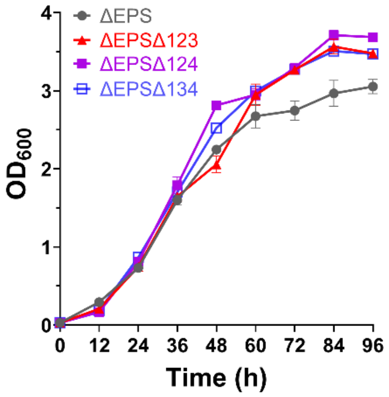

Figure S1. OD_{600} curves of *H. mediterranei* strains cultured in AS-168 medium. ΔEPSΔ123, ΔEPSΔ*oriC1*Δ*oriC2*Δ*oriC3*; ΔEPSΔ124, ΔEPSΔ*oriC1*Δ*oriC2*Δ*oriC4*; ΔEPSΔ134, ΔEPSΔ*oriC1*Δ*oriC3*Δ*oriC4*.

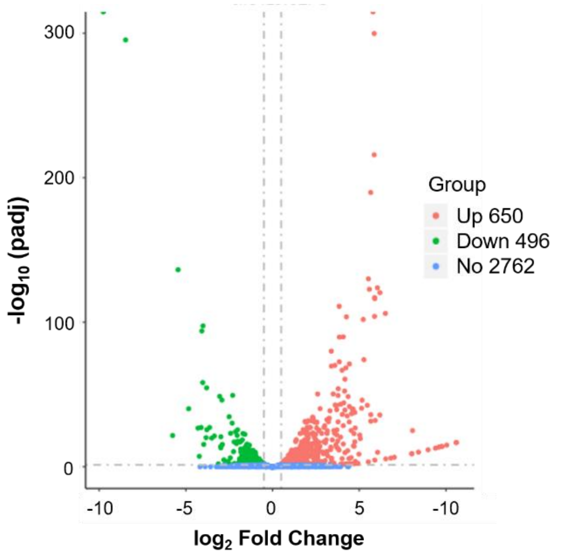

Figure S2. Volcano plot of DEGs (differentially expressed genes) distribution. Red plots, upregulated genes; green plots, downregulated genes; blue plots, non-significantly different expression genes.

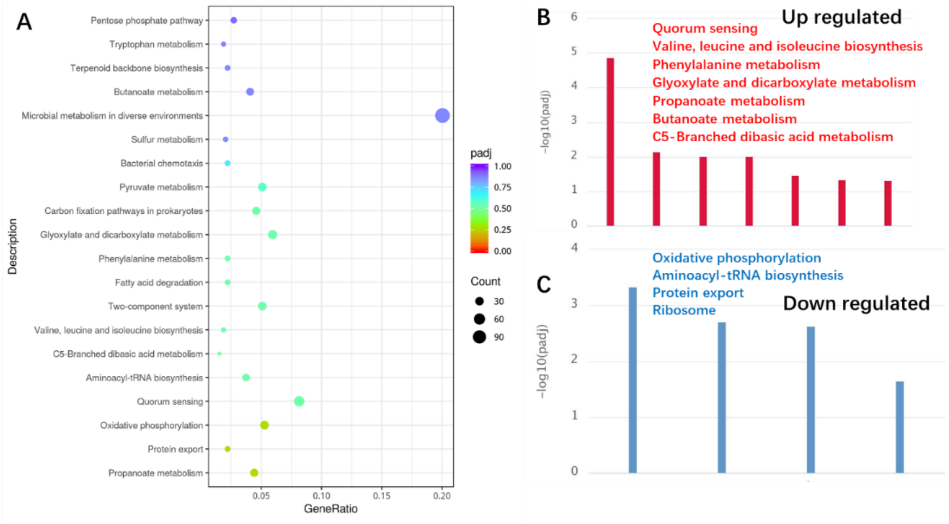

Figure S3. **KEGG enrichment.** (A) Scatter plot of KEGG enrichment analysis. (B) Significantly upregulated pathways. (C) Significantly downregulated pathways. In (B) and (C), *padj* is set as 0.05.

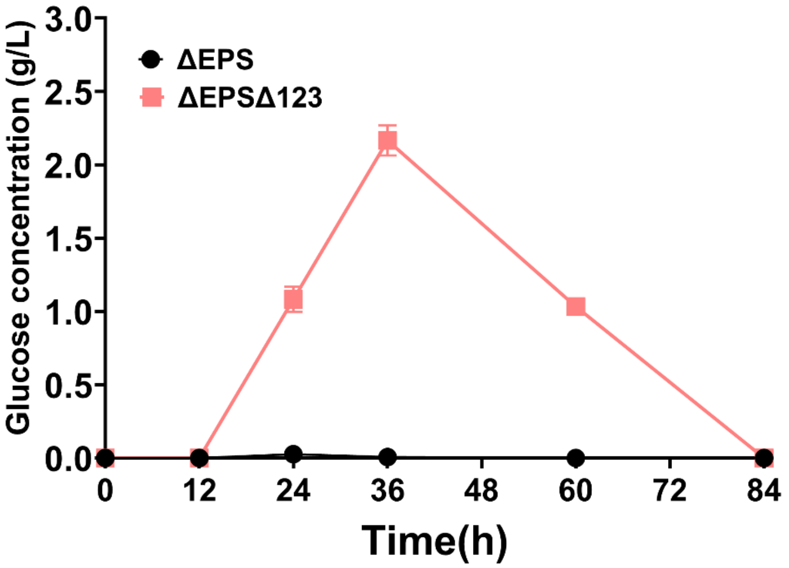

Figure S4. Glucose concentration of ΔEPS and ΔEPSΔ123 in MS medium. The mutants of *H. mediterranei* were cultured in MS medium with 20 g/L starch as the carbon source. All data are expressed as means ± standard deviations of three biological replicates. ΔEPSΔ123, ΔEPSΔ*oriC1*Δ*oriC2*Δ*oriC3*.

References

1. Sambrook, J., Fritsch, E.F. and Maniatis, T. 1989. Molecular Cloning: A Laboratory Manual.
2. Liu, H., Han, J., Liu, X., Zhou, J. and Xiang, H. 2011. Development of *pyrF*-based gene knockout systems for genome-wide manipulation of the archaea *Haloferax mediterranei* and *Haloarcula hispanica*. J Genet Genomics **38**, 261-269.
3. Yang, H., Wu, Z., Liu, J., Liu, X., Wang, L., Cai, S. and Xiang, H. 2015. Activation of a dormant replication origin is essential for *Haloferax mediterranei* lacking the primary origins. Nat Commun **6**, 8321.
4. Zhao, D., Cai, L., Wu, J., Li, M., Liu, H., Han, J., Zhou, J. and Xiang, H. 2013. Improving polyhydroxyalkanoate production by knocking out the genes involved in exopolysaccharide biosynthesis in *Haloferax mediterranei*. Appl Microbiol Biotechnol **97**, 3027-3036.
